# Heterologous expression and mutagenesis of recombinant *Vespa affinis* hyaluronidase protein (rVesA2)

**DOI:** 10.1590/1678-9199-JVATITD-2019-0030

**Published:** 2019-12-05

**Authors:** Prapenpuksiri Rungsa, Piyapon Janpan, Yutthakan Saengkun, Nisachon Jangpromma, Sompong Klaynongsruang, Rina Patramanon, Nunthawun Uawonggul, Jureerut Daduang, Sakda Daduang

**Affiliations:** 1 Protein and Proteomics Research Center for Commercial and Industrial Purposes (ProCCI), Department of Biochemistry, Faculty of Science, Khon Kaen University, Khon Kaen 40002, Thailand; 2 Division of Pharmacognosy and Toxicology, Faculty of Pharmaceutical Sciences, Khon Kaen University, Khon Kaen 40002, Thailand; 3 Faculty of Science, Nakhon Phanom University, Nakhon Phanom, 48000, Thailand; 4 Centre for Research and Development of Medical Diagnostic Laboratories, Faculty of Associated Medical Sciences, Khon Kaen University, Khon Kaen, Thailand

**Keywords:** Vespa affinis, Hyaluronidase, Wasp, Venom, Structure analysis, Modelling, Cloning, Protein expression

## Abstract

**Background:**

Crude venom of the banded tiger wasp*Vespa affinis* contains a variety of enzymes including hyaluronidases, commonly known as spreading factors.

**Methods:**

The cDNA cloning, sequence analysis and structural modelling of *V. affinis* venom hyaluronidase (VesA2) were herein described. Moreover, heterologous expression and mutagenesis of rVesA2 were performed.

**Results:**

*V. affinis* venom hyaluronidase full sequence is composed of 331 amino acids, with four predicted *N*-glycosylation sites. It was classified into the glycoside hydrolase family 56. The homology modelling exhibited a central core (α/β)_7_ composed of Asp107 and Glu109, acting as the catalytic residues. The recombinant protein was successfully expressed in *E. coli* with hyaluronidase activity. A recombinant mutant type with the double point mutation, Asp107Asn and Glu109Gln, completely lost this activity. The hyaluronidase from crude venom exhibited activity from pH 2 to 7. The recombinant wild type showed its maximal activity at pH 2 but decreased rapidly to nearly zero at pH 3 and was completely lost at pH 4.

**Conclusion:**

The recombinant wild-type protein showed its maximal activity at pH 2, more acidic pH than that found in the crude venom. The glycosylation was predicted to be responsible for the pH optimum and thermal stability of the enzymes activity.

## Background

Hyaluronidase is an enzyme family that catalyze the hydrolysis of hyaluronic acid (HA) and several other glycosaminoglycan constituents of the extracellular matrix of vertebrates. It is often found in all types of animal venom [1, 2, 3]. Venom toxin cocktails comprise high-molecular weight molecules, such as phospholipases A (PLA), hyaluronidases, antigen 5 and acid phosphatase, low-molecular weight compounds and peptides such as hemolytic peptides, antimicrobial peptides, and amines [4, 5, 6, 7, 8]. In animal venoms, hyaluronidases degrade hyaluronic acids in extracellular matrix and are generally referred to as “spreading factors” that enhance envenomation by increasing the absorption and diffusion rate of systemic venom toxins in the circulation of prey [9]. Venom hyaluronidases have also been identified as major allergens of scorpions, bees, hornets and wasps, which can induce serious and occasionally fatal systemic IgE-mediated anaphylactic reactions in humans [1, 10-12]. Several studies have reported the purification and characterization of venom hyaluronidases from spider [13, 14], scorpion [15, 16], conus [17], snake [18, 19, 20], freshwater stingray [9] and wasp [21].

The hyaluronidase from Hymenoptera venom is relatively conserved. Hymenoptera stings represent one of the three major causes of anaphylaxis worldwide [5], including serious symptoms after envenomation [22, 23]. *Vespa affinis*, the tiger wasp, are mostly found in the Asia-Pacific region, including Thailand. The nests of *V. affinis* are typically located in the trees of forests near human habitats, which results in a record-breaking number of stinging accidents every year [24]. 

The venom of *V. affinis* is lethal. Sukprasert et al. [25] reported a paralytic dose (PD_50_) of approximately 12.2 µg/g of body weight in crickets (*Gryllus* sp.). The major venom allergen proteins are PLA, with 100% allergenicity, and hyaluronidase, with 53.3% allergenicity [24]. Additionally, the proteomic analysis of *V. affinis* venom performed by Rungsa et al. [26] detected venom hyaluronidases, which are major venom proteins. Anti-hyaluronidase serum inhibited or delayed the occurrence of large tissue damage, potentially allowing a more efficient clinical management of the victim [27, 28]. 

In the present study, the cDNA encoding *V. affinis* hyaluronidase was sequenced. The amino acid sequences were also deduced. The *in silico* prediction of its higher-level protein structures was performed. A mutant type with amino acid substitutions at the catalytic site was produced to elucidate their functions. The activity of recombinant mutant type was comparatively characterized in relation to that of the wild-type protein.

## METHODS

### Materials

The worker wasp *V. affinis* was obtained from Nakornphanom Province, Thailand. The venom glands were dissected and kept at -80°C. The bacterial strains and the ImPromII Reverse Transcription System kit was acquired from Invitrogen Life Technologies (USA). We purchased the pET32a expression plasmid from Novagen (USA).

The present study was approved by the Animal Ethics Committee of Khon Kaen University based on the Ethics for Animal Experimentation of the National Research Council of Thailand (Reference. 0514.1.12.2/1).

### Protein biochemistry

One-dimensional polyacrylamide gel electrophoresis was performed following standard methods, using 13% (w/v) separating gels and 4% (w/v) stacking gels. The low molecular-weight marker (GE Healthcare, USA) was used as the protein standard. The gel was separated at 150 volts for 80 min. After separation, the gel was stained with Coomassie blue R-250 staining solution. The protein band was excised from the 13% SDS-PAGE gel. An in-gel digestion was performed according to the previous description from Rungsa et al. [29]. The gel was digested using trypsin solution (20 ng trypsin in 50% ACN/10% ammonium bicarbonate) following a standard method described by the Research Instrument Center, Khon Kaen University, Thailand. The sample was analyzed with a nano-LC (EasynLC II, Bruker Daltonics, USA) coupled to an ion trap mass spectrometer (Amazon Speed ETD, Bruker, USA) equipped with an ESI nano-sprayer. LC-MS/MS spectra were analyzed using Compass Data Analysis v. 4.0. Compound lists were exported as Mascot generic files (mgf) for further analysis in the Mascot program [26].

### **Cloning and isolation of cDNA encoding *V. affinis* hyaluronidase using PCR techniques**

Total RNA was extracted from the *V. affinis* venom gland using TRIzol® reagent (Invitrogen Life Technologies, USA). First-strand DNA synthesis was performed using a RevertAid First stand cDNA synthesis Kit (Thermo Scientific, USA) following the manufacturer’s instructions for the PCR amplification of encoded sequences. The amplification of hyaluronidase genes was performed using master mix reagent kits with Taq DNA polymerase (Vivantis, Malaysia). The primers were described by Rungsa et al. [27, 30]. The 3'rapid amplification of cDNA ends (3'RACE) was carried out according to the kit’s instruction manual (Invitrogen Life Technologies, USA) using gene-specific primers and AUAP universal primers. The PCR products were purified using GenepHlow Gel Extraction kits (Geneaid, Taiwan) and cloned into a pGEM®-T easy vector (Promega, USA) for sequencing [29, 31].

### Sequence alignments, the prediction of secondary structure and homology modelling

The *V. affinis* hyaluronidase sequence (VesA2) was analyzed using FinchTV and BLAST (http://www.ncbi.nlm.nih.gov/BLAST/), and a multisequence alignment was carried out using Multiple Sequence Alignment Clustal Omega (https://www.ebi.ac.uk/Tools/msa/clustalo). The ExPasy tool was used to translate the sequence (https://web.expasy.org/translate/). The protein sequence was examined with the SWISS-MODEL for three-dimensional structure prediction. The structure was investigated with the PDB viewer program (PDB; http://swissmodel.expasy.org/) and Chimera software (https://www.cgl.ucsf.edu/chimera/download.html). The stereochemical quality validation of the model was confirmed using Ramachandran plot. The *N*-glycosylation prediction was performed using the CBS prediction severs (http://www.cbs.dtu.dk/services/NetNGlyc/). The free web server DiANNA (http://clavius.bc.edu/~clotelab/DiANNA/) was used to predict the formation of disulfide bonds.

### **Cloning and expression of the recombinant gene in *E. coli***

The wild-type *V. affinis* hyaluronidase was amplified by polymerase chain reaction (PCR). The forward and reverse primers contained *Kpn* I and *Not* I restriction sites, respectively. The PCR-amplified products were sequentially subjected to 1.2% agarose gel electrophoresis, double digestion with *Kpn* I/*Not* I restriction enzymes and cloning into a pre-digested pET32a expression vector following the manufacturer’s instructions. The constructs were transformed into *Escherichia coli* BL-21 (DE3) chemically competent cells, plated on Luria Bertani (LB) agar plates containing ampicillin and incubated at 37ºC overnight. The colony was verified by colony PCR and analyzed by DNA sequencing using an Automated PCR sequencer (First base, Malaysia) [32].

### Site-directed mutagenesis

Site-directed mutagenesis was carried out using splicing by overlapping extension PCR to create the mutant *V. affinis* hyaluronidase following previous studies [33]. The mature *V. affinis* hyaluronidase in the pGEM-T easy vector was used as the template for mutagenesis. The two sites were chosen for PCR-based site-directed mutagenesis (Table 1). The *Pfu* DNA polymerase was used for the amplification the recombinant mutant type. The mutant VesA2 was poly-A-tailed by *Taq* polymerase and cloned into the pGEM-T easy vector. The positive clones were verified by colony PCR. The cDNA encoding hyaluronidase was subcloned into pET32a.

**Table 1.** Gene-specific primers that were designed in this study

F-*Kpn* I primer	5'-GGTACCTCCGAGAGACCGAAAAAAG-3'
R-*Not* I primer	5'-GCGGCCGCAGTTAACGGCTTCTGTCA-3'
F-mutant	5'-GGTATAATCAACTTTCAAAGATGGAGA-3'
R-mutant	5'-TCTCCATCTTTGAAAGTTGATTATACC-3'

### Small-scale expression and optimization of the expression conditions

The *E. coli* cells containing the recombinant *V. affinis* hyaluronidase gene (wild-type and mutant) were grown in 5 mL LB liquid medium containing 50 µg/mL ampicillin at 37°C overnight. A total of 50 µL of pre-cultured cells was added to 5 mL fresh LB liquid medium until the OD_600_ reached 0.4-0.6. The isopropyl-β-D-thiogalactopyranoside (IPTG) concentration, induction time and temperature for the expression of foreign proteins in *E. coli* BL-21(DE3) were optimized. Under 15 and 37°C, IPTG was added to each fresh subculture (OD_600_ = 0.5) with different final concentrations (0, 0.1, 0.2, 0.3, 0.4, 0.5, 1 and 1.5 mM) and was incubated for additional 24 hours for the optimization of the IPTG concentration. For the optimization of the induction time, subcultures were incubated for additional times (non-induction, 4, 6, 8, 10 hours and overnight) with optimal conditions of the IPTG concentration and induction temperature. The temperature induction was performed at various temperatures (15°C and 37°C). All liquid subcultures were collected and then mixed with 2x solubilizing solution (v/v: 1/1) [0.5 M Tris-HCl, pH 6.8, 10% (v/v) glycerol, 10% (w/v) sodium dodecyl sulfate (SDS) and 1% (w/v) bromophenol blue] and heated at 100°C for 10 min for analysis using SDS-PAGE to choose the optimal culture parameters.

### Up-scale expression and purification of recombinant proteins

For the up-scale expression of recombinant *V. affinis* hyaluronidase, the optimal expression conditions were used. The recombinant *V. affinis* hyaluronidase was cultured in 10 mL LB liquid medium containing 50 µg/mL ampicillin at 37°C overnight. Fresh LB liquid medium (1 L) containing 50 µg/mL ampicillin was incubated with 10 mL overnight culture until the OD_600_ reached 0.3-0.8, and IPTG was added at the optimized concentration. The *E. coli* cells were harvested by centrifugation at 5000 × g for 10 min at 4°C, suspended in 30 mL of lysis buffer (50 mM Tris-HCl, pH 8, 100 mM NaCl, 1 mM DTT, and 0.1 mg/mL lysozyme) and lysed. 

The recombinant protein was detected in the insoluble fraction. The cell pellet was washed using 20 mM Tris-HCl, pH 8 and 2 M urea. Then, it was solubilized in 20 mM Tris-HCl, pH 8 and 1 mM DTT containing 4 M urea with stirring at room temperature for 3 hours. The soluble fraction was collected after centrifugation (10000 × g, 30 min) and kept at -20°C until use. The soluble fraction was dialyzed using reducing urea concentrations (20 mM Tris-HCl, pH 8, 10% glycerol and 2 M or 0 M urea, respectively). The refolded protein was purified using His-gravitrap column (GE healthcare, USA) following manual instruction in 20 mM Tris-HCl, pH 8, under a step-wise imidazole concentration. The purified protein was concentrated using Centricon® 30 kDa filters and used for the enzymatic testing.

### Hyaluronidase activity assay

The zymographic gel hyaluronidase activity assay was performed using 13% SDS-PAGE containing 0.5 mg/mL hyaluronic acid (Sigma, USA) as a substrate. The gel was incubated in 3% Triton X-100 for 1 hour, transferred to the hyaluronidase assay buffer (0.15 M NaCl in 0.1 M formate buffer pH 3.7) for 16 hours. After that, gels were stained in Alcian Blue solution (0.5% Alcian Blue in 3% acetic acid) and destained in 7% acetic acid until the clear band was appeared [34].

A turbidity hyaluronidase activity assay was performed in a reaction mixture containing 100 μg crude venom/fraction, 0.5 mg/mL hyaluronic acid and 0.2 M formate buffer, pH 3, containing 0.15 M NaCl and was incubated for 30 min at 37°C. A 2% cetyltrimethylammonium bromide (CTAB) (w/v) solution containing 2.5% NaOH (w/v) was used to stop the reaction, and the absorbance was measured at 405 nm. One unit of hyaluronidase enzyme activity has been defined as the quantity of the enzyme that reduce the turbidity equal to one unit of international standard preparation after incubating with substrate at 37°C for 30 min at pH 4 [35].

### The effect of pH and temperature on the activity assay

The optimum pH of the recombinant enzyme was determined using 50 mM formate buffer (pH 2-4), 50 mM sodium acetate buffer (pH 5-6) and 50 mM Tris-HCl buffer (pH 7-10). The pH stability was investigated by preincubating the enzyme at each pH for 30 min at 37°C and then measuring the residual activities. The optimum temperature was analyzed over a range of 30-80°C, and the thermostability was investigated by preincubating the enzyme in the absence of substrate for the indicated times at 50 and 60°C and then measuring the residual activities [27].

## Results

### Sequencing and structural analysis

**Figure 1.**
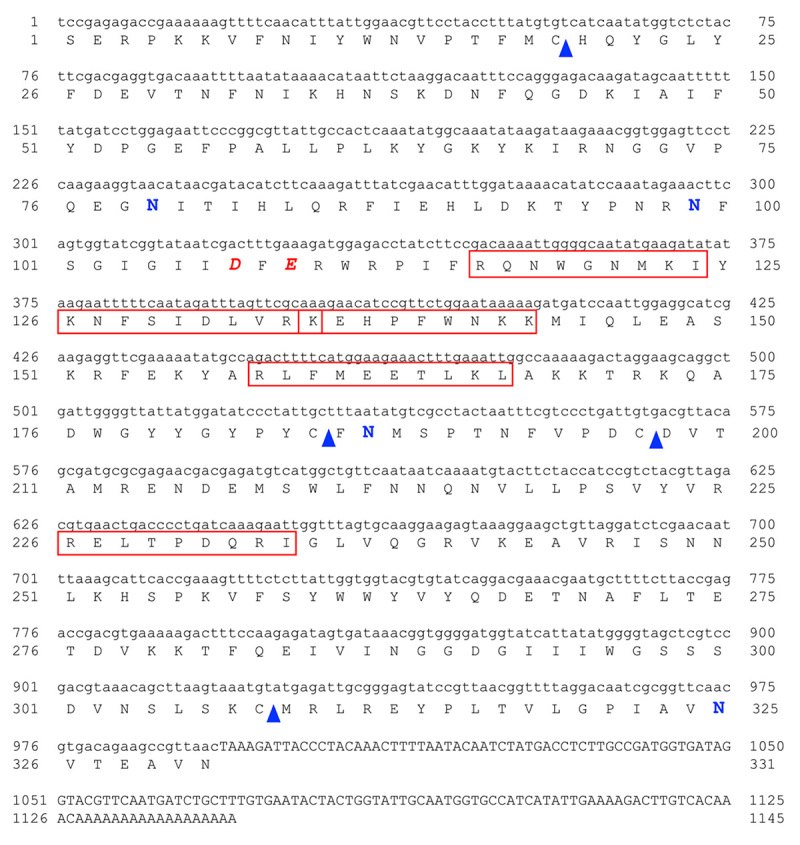
**The complete nucleotide and predicted amino acid sequences of *Vespa affinis* venom hyaluronidase (VesA2).** The red italic capital letters (“D” for Asp and “E” for Glu) indicate the catalytic residues of the active sites. Four cysteine residues (“C”) - indicated with blue triangles - form two disulfide bonds. Based on the *in silico* prediction, the two bonds were C19-C185 and C197-C308. The four predicted *N*-glycosylation sites (asparagine, “N”) are indicated with blue letters. The peptides from the LC-MS/MS analysis are shown in the red boxes.

The full length of the wild-type *V. affinis* venom hyaluronidase gene (VesA2) was determined with classical strategies, using mRNA from the venom gland as the template. The full nucleotide sequence of VesA2 was 1145 bp in length and had 152 bp in the 3'untranslated region (3'UTR). The prediction revealed that the primary sequence of the wild-type *V. affinis* hyaluronidase polypeptide (VesA2) contained 331 amino acids (Figure 1). The theoretical average mass was 39.0487 kDa whereas the theoretical isoelectric point (pI) was 9.16. Four potential *N*-glycosylation sites (Asn-Xaa-Thr/Ser, where Xaa is any amino acid residue except proline), Asn79, Asn99, Asn187 and Asn325, and two disulfide bridges, C19-C185 and C197-C308, were predicted. The amino acid residues Asp and Glu, which are commonly found in active sites, usually acting as catalytic residues, were observed at positions 107 and 109, respectively [36].

**Figure 2.**
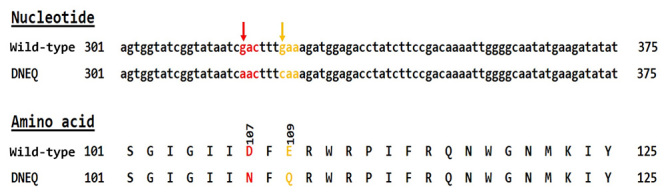
**The amino acid sequence comparison of wild-type and mutant VesA2.** The nucleotide guanine 319 (g319) and guanine 225 (g325) in the wild-type sequence were changed to adenine (a319) and cytosine (c325) in the mutant sequence. These replacements caused the amino acid aspartic acid (Asp 107) to change to asparagine (red letters), and glutamic acid (Glu109) changed to glutamine (yellow letters).

The generation of a mutant protein with the substitution of Asp107 and Glu109 to Asn107 and Gln109 was carried out with site-directed mutagenesis strategies. Oligonucleotide primers containing the restriction enzyme cutting sites for *Kpn* I and *Not* I were synthesized and used for PCR techniques to obtain Asp107Asn and Glu109Gln substitution mutations (Table 1). The mutations were successfully produced. The subsequent nucleotide sequencing, prediction and NCBI-BLAST search confirmed the presence of these substitutions (Figure 2). The predicted amino acid sequences showed high homology to the *V. affinis* hyaluronidase sequences from LC-MS/MS analysis [26].

### Multiple sequence alignment and homology modelling

**Figure 3.**
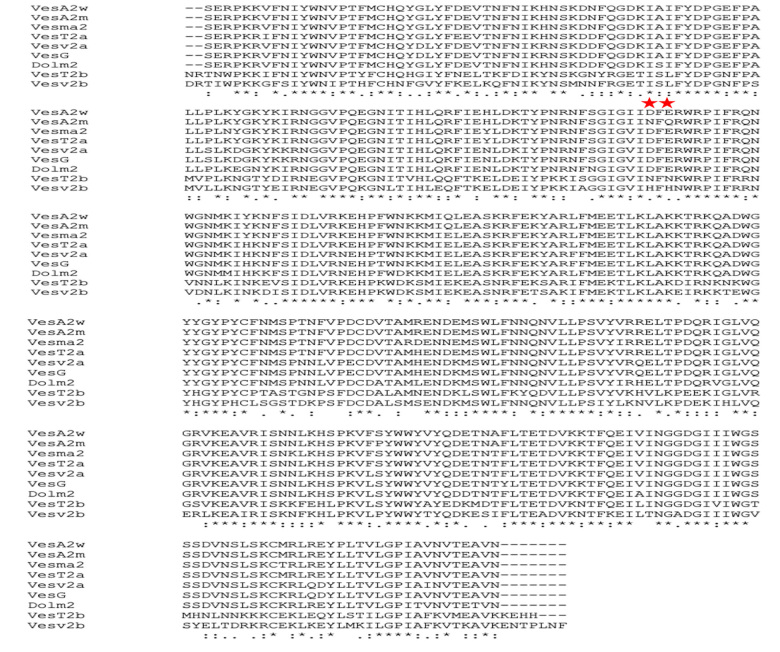
**Multiple alignments among the primary sequence of wasp venom hyaluronidases.** Stars designate identical residues. Colons and dots indicate similar residues. The catalytic residues (“D” and “E”) are indicated with red stars. VesA2w is the wild-type VesA2. VesA2m is the mutant VesA2. Vesma2 (*Vespa magnifica*), VesT2 (*Vespa tropica*) Vesv2 (*Vespula vulgaris*), Dolm2 (*Dolichovespula maculata*), and VesG (*Vespa germanica*) are shown. The small “a” and “b” of Vesv2 and VesT2 indicate “active” and “inactive” hyaluronidases, respectively.

The wild-type VesA2 sequence alignment showed high homology to active wasp venom hyaluronidase: 96.67% *Vespa tropica* (VesT2a), 96.07% *Vespa magnifica* (Ves ma2*)*, 90.05% *Dolichovespula maculata* (Dol m2), 91.54% *Vespa germanica* (Ves g) and 91.23% *Vespula vulgaris* (Ves v2a). The wild-type VesA2 showed less homology to those in inactive forms of wasp venom hyaluronidase: 58.61% *Vespula vulgaris* (Ves v2b) and 61.93% VesT2b (Figure 3).

**Figure 4.**
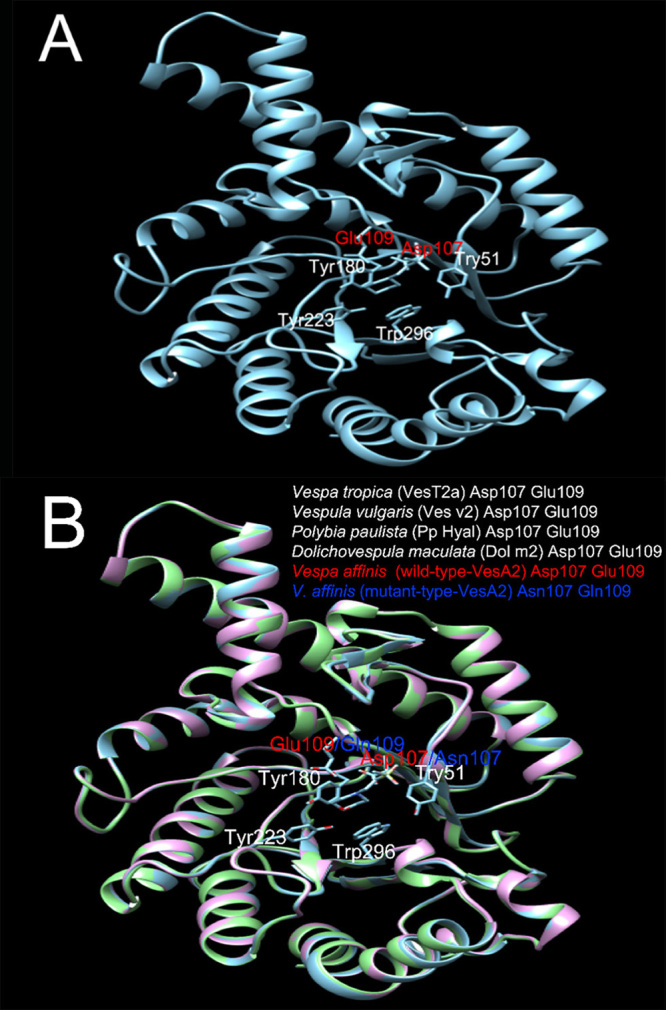
**The ribbon representation of the predicted three-dimensional structural modelling of VesA2.** (A) The structure of wild-type VesA2 (blue ribbon) using *Vespula vulgaris* venom hyaluronidase (PDB ID: 2ATM) as the template. (B) The superimposition of wild-type VesA2 (blue), mutant VesA2 (green) and 2ATM (*Vespula vulgaris* venom hyaluronidase, pink ribbon). The catalytic residues in the active sites are indicated (Asp107 and Glu109). The mutant strains contained Asn107 and Gln109. The labels on the top show the catalytic resides of the venom hyaluronidases from the databases.

The constructed three-dimensional structure model of VesA2 using the SWISS-MODEL protein homology modelling server was performed based on two templates with structures perfectly clarified from crystallography. Those templates were hyaluronidases from wasp venom (*V. valgaris*; Ves v 2; 2ATM) (Figure 4) and bee venom hyaluronidase (*Apis mellifera*; Api m2; 2J88) (data not shown) [36, 37]. The Ves v 2 and Api m2 templates showed 91.23 and 52.74% sequence identity, respectively, to VesA2. Bee venom hyaluronidase, Api m2, was composed of three parts: hyalurononglucosaminidase A, Fab B and Fab C. VesA2 showed high similarity to the hyalurononglucosaminidase A part [38]. 

From Ramachandran plot, phi and psi conformation angels of VesA2 backbone for each residues of amino acid were displayed (data not shown). Plot statistics of the model exhibited 90.4% of the residues in favored regions, 9.3% in additional allowed regions, 0% in generously allowed regions and 0.4% in disallowed regions [39]. VesA2 is composed of seven α-helices and seven β-sheets belonging to the glycoside hydrolase family 56 (E.C. number 3.2.1.35). Figure 4 shows that the position and orientation of the catalytic site and other conserved residues coincide fairly well. The substrate-adjacent residues fell into three regions. The residue contact substrate can be presumed to be involved in binding and substrate recognition. Asp107 and Glu109 are common catalytic residues of Hymenoptera venom hyaluronidase that are found in the active sites. Tyr51, Tyr180, Tyr223 and Typ296 are nearby residues that function proximally to the cleavage point of the substrate and are likely to have been in contact with a transition state and/or the released portion of the cleaved HA chain.

### Recombinant wild-type and mutant VesA2 expression

The mature recombinant VesA2 was subcloned into the pET32a expression vector containing a 6xHis tag and a thioredoxin fusion at the N-terminus. These tags are useful for recombinant protein expression and solubilization. The peptide mass fingerprints from the LC-MS/MS analysis subsequently searched by Mascot search of wild-type and mutant type rVesA2 revealed high similarity to the hyaluronidase of *V. magnifica* venom (Table 2).

**Table 2.** Identification of wild-type and mutant recombinant hyaluronidase (VesA2) of *Vespa affinis* venom

Recombinant protein	Peptide sequences	XC score	Species
Wild type	R.ELTPDQR.I R.QNWGNMK.I K.EHPFWNK.K K.NFSIDLVR.K R.LFMEETLK.L R.RELTPDQR.I R.LFMEETLK.L	426	*Vespa magnifica*
Mutant type	R.QNWGNMK.I K.EHPFWNK.K K.NFSIDLVR.K R.LFMEETLK.L R.NGGVPQEGNITIHLQR.F K.TFQEIVINGGDGIIIWGSSSDVNSLSK.C	243	*Vespa magnifica*

**Figure 5.**
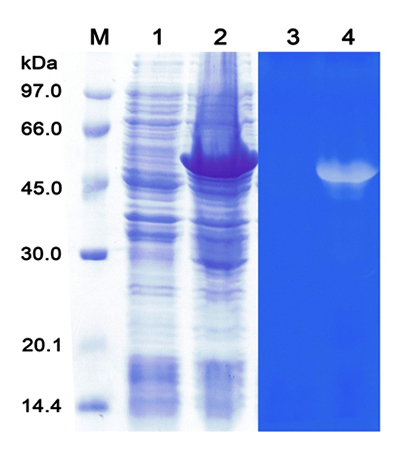
**Wild-type recombinant VesA2 (rVesA2) expression in *E. coli* BL-21 (DE3).** Wild-type rVesA2 expression. Lanes 1 and 2 were analyzed by SDS-PAGE, and lanes 3 and 4 were assayed for hyaluronidase activity.

**Figure 6.**
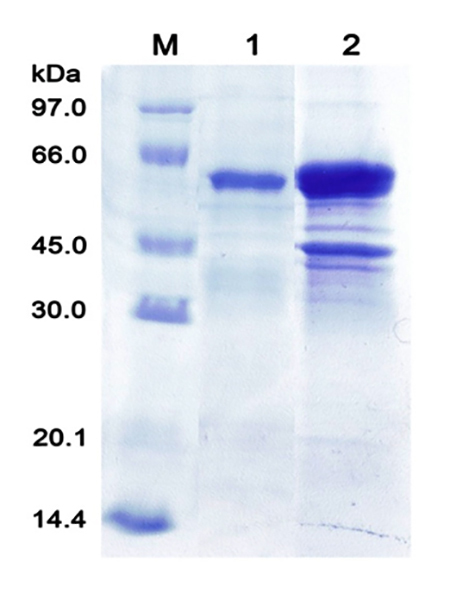
**Solubility of wild-type and mutant VesA2.** The wild-type rVesA2 was solubilized in 4 M urea (lane 1). The mutant protein was solubilized in 6 M urea (Lane 2)

**Figure 7.**
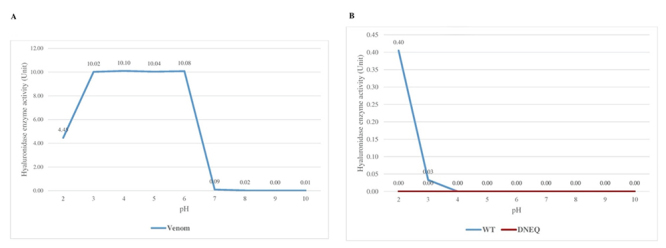
**The optimal pH of (A) *V. affinis* venom hyaluronidase and (B) wild-type and mutant rVesA2.** **(A)** The crude venom showed relatively high hyaluronidase activity at low and neutral pH. **(B)** The wild-type rVesA2 showed activity at pH 2-3 and completely lost its activity at pH 4 (blue line). The mutant rVesA had no enzymatic activity (red line).

**Figure 8.**
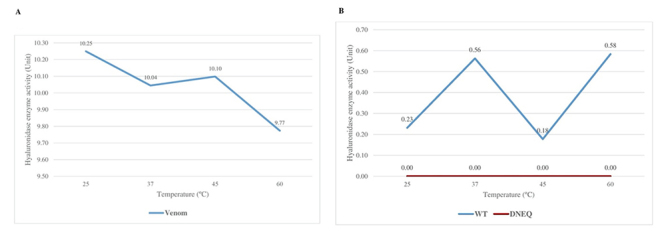
**The optimal temperature of (A) *V. affinis* venom hyaluronidase and (B) wild-type and mutant rVesA2.** The optimal temperatures were tested in buffer at pH 2. **(A)** The crude venom showed high hyaluronidase activity at 25 to 60°C and completely lost hyaluronidase activity at 70°C. **(B)** The wild-type rVesA2 showed activity at 25 to 60°C (blue line). The mutant had no hyaluronidase activity (red line).

The expression conditions of the wild-type and mutant rVesA2 for maximal over-expression were the induction with 0.1 mM IPTG at 37°C for 4 hours. The wild-type protein exhibited high zymographic gel hyaluronidase activity (Figure 5), whereas the mutant type completely lost this activity (data not shown). The overexpressed protein band from heterologous expression in *E. coli* was approximately 59 kDa on an SDS denaturing gel, corresponding to a transparent band in the blue background of the zymographic gel of the hyaluronidase activity assay. The size (approximately 59 kDa) was larger than the theoretical mass (~39 kDa) and was the summation of the VesA2 gene and tags. However, the process of solubility with sonication revealed that the recombinant proteins were insoluble in the aqueous-based buffer commonly known as inclusion bodies (data not shown). The solubility test by SDS-PAGE showed that rVesA2 mainly appeared in the insoluble fraction. To increase the solubility of the recombinant proteins, 4 and 6 M urea were used (Figure 6). After the renaturation of recombinant wild type and mutant type, the hyaluronidase activity was recovered based on an analysis using turbidity hyaluronidase activity assay (Figure 7B and Figure 8B). The recovery yields of both recombinant types ranging from 13.0 to 22.5 mg per 1 liter of culture media. During induction, temperature may be a variable. Therefore, the temperature was varied from 15 to 37°C, and the inclusion bodies remained a problem (data not shown).

### Hyaluronidase activity of the wild-type and mutant rVesA2

A hyaluronidase activity test with a turbidity assay showed that the *V. affinis* venom had activity at pH ranging from 2 to 10, with maximal activity at pH 6 (Figure 7A). The recombinant rVesA2 had activity around pH 2 to 3, with an optimal pH at 2. The mutant protein completely lost its activity from pH 2 to 10 (Figure 7B). The optimal temperature for venom hyaluronidase was 25°C but the protein was still active at 60°C (Figure 8A). The activity was completely lost after incubation at 70°C for 30 mins (data not shown).

## Discussion

Wasp venoms are complex mixtures of biologically active proteins and peptides [5, 21, 25, 26, 40]. Interestingly, Rungsa et al. [27] reported that the antivenoms or inhibitors of the hyaluronidase enzyme increased the venom toxicity. These enzymes are described as spreading factors that facilitate the distribution of other venom components through tissues, causing highly potent and fast acting venom toxicity [1, 20]. In addition, the high enzymatic activity of these proteins are from crude venom found in the venom gland, which has a low protein quantity. However, the molecular cloning of *V. affinis* hyaluronidase was performed to attempt to produce the recombinant protein, which is similar to that of the natural sources. The gene encoding VesA2 was cloned into *E. coli* using the pET32a vector. The double Asp107Asn and Glu109Gln mutant protein was prepared. Both the wild-type and mutant proteins were expressed and refolded. The rVesA2 wild-type and mutant proteins were assayed for hyaluronidase activity with a turbidity assay, including evaluating their optimal temperature and optimal pH.

In a previous report, hyaluronidase from the venom of *V. affinis* had a molecular weight of approximately 43 kDa based on 2D-PAGE and LC-MS/MS analysis [26]. The sequence analysis of VesA2 showed a molecular weight of approximately 39.04 kDa. The 43-kDa mass protein was 4 kDa higher than the theoretical mass and was predicted to be the result of the carbohydrate moiety attachment. Hymenoptera venom-derived hyaluronidases are always post-translationally glycosylated, which causes extensively higher allergenic properties in the venom [36, 37]. The cross-reactivity carbohydrate determinant (CCD) contributed the immunogenicity in Hymenoptera venom, such as bee venom or wasp venom [37, 41]. The Hymenoptera stings represent one of the three major causes of anaphylaxis worldwide [5].

Recombinant Ves v 2, originally from *V. vulgaris* venom, a representative wasp venom hyaluronidase, was modelled and structurally analyzed [36]. Those proteins, including VesA2, were classified in the glycoside hydrolase family 56 (E.C. number 3.2.1.35) [42, 43]. Based on the three-dimensional model structure, VesA2 is composed of seven β-sheets and seven α-helices with a central core (α/β)_7_ [1]. Four cysteines are the most conserved residues among active venom hyaluronidase since they produce two disulfide bridges that stabilize their three-dimensional structure. 

Hymenoptera venom hyaluronidase is relatively conserved, with molecular weights ranging from 33 to 40 kDa. The molecular weight of this venom protein was dependent on the CCD to the polypeptides [44, 45]. These proteins are *N*-glycosylated, which exerts a direct influence on the immunogenicity [41]. The four residues for VesA2 that were *N*-linked were Asn79, Asn99, Asn187 and Asn325. Asn79 and Asn99 corresponded to the native Ves v 2 [36]. Aspartate and glutamate residues commonly serve as the catalytic residues of Hymenoptera hyaluronidase. Marković-Housley et al. [37] reported that Asp111 and Glu113 of bee venom hyaluronidase were proton donors for catalysis. These residues corresponded to Asp107 and Glu109 in many kinds of wasp venom, including Ves v 2 and VesA2 from this study. Aspartate and glutamate act as proton donors, whereas the *N*-acetyl carboxyl groups of the substrate hyaluronic acid (HA) act as nucleophilic bases [37, 46]. Meanwhile, four residues, including tyrosine at position 51, 180, 223 and tryptophan at 296, are nearby residues that function proximally to the cleavage point of the substrate and are likely in contact with a transition state and/or the released portion of the cleaved HA chain.

Previous studies verified the catalytic residues of wasp venom hyaluronidases using *in silico* structural analysis. To confirm two catalytic residues (Asp107 and Glu109) using *in vitro* system*,* the present work constructed the mutant type of VesA2 by the double point mutation of Asp107 and Glu109 in order to investigate their individual contributions to the enzymatic activity. The structure of Asn and Gln are basically similar to Asp and Glu, but the R-side chains are converted from acid to the amide groups. The mutant with the double point mutation completely lost its enzymatic activity. These phenomena have been reported previously [46]. The Asn and Gln lack dissociated protons, therefore, are incapable of substitution as general acid/base [47].

For gene expression, the attempts to obtain a recombinant hyaluronidase from the venom of social Hymenoptera in *E. coli* have been previously reported [12]. These recombinant proteins were not toxic to host cell, because the recombinant fused to a fusion partner in heterologous hosts to neutralize their innate toxicity and increase their expression levels [48]. From gene expression, inclusion bodies in the bacterial system seem unavoidable [49]. This study tried to use the pET32a vector with a thioredoxin (Trx) tag to promote the solubility of protein targets in the cytoplasm of *E. coli* and facilitate the formation of disulfide bonds [50] However, the wild-type and mutant recombinant VesA2 were expressed in inclusion bodies. 

The recombinant protein from *Polybia paulista* venom was insoluble. However, the recombinant showed a 100% pattern of cross reactivity with the native protein after detection using specific IgEs from patient sera that recognized the *P. paulista* venom. These results demonstrated the high degree of sensitivity and specificity of the IgE antibodies to the hyaluronidase allergen. The primary structures of both proteins were unchanged, resulting in a similar secondary structure. This indicates that the venom hyaluronidase may be the primary factor responsible for triggering allergic symptoms that are caused after accidents with this wasp [12]. The primary structure of both proteins has been confirmed by partial amino acid sequence analysis by mass peptide fingerprinting using LC-MS/MS. These proteins exhibited a significantly high homology to venom hyaluronidase from other Hymenoptera, such as *V. magnifica*, *V. tropica* and *V. affinis* [26]. 

The mutant rVesA2 showed the same percentage of homology to the wild-type because only two nucleotides had been substituted to form a double point mutation. The insoluble protein showed activity on a hyaluronidase zymographic gel [34]. The stepwise reduction of the urea concentration by dialysis was required to recover the enzymatic activity after the activity was assayed with the hyaluronidase turbidimetric method. However, for the mutant rVesA2, the substitution of Asp to Asn and Glu to Gln in the mutant prominently affected the protein activity. The mutant protein completely lost this activity. Asp107 and Glu109 are theoretically the most important catalytic residues in the active site of hyaluronidase.

Venom hyaluronidases are active in a variety of pH ranges. In 2013, Cavallini et al. [51] classified the enzymes into two groups. The acid hyaluronidases are active in the pH range from 2 to 4. The other group, neutral hyaluronidases, are active from pH 5 to 6 [51]. The enzymes in crude venom and recombinant wild-type VesA2 tend to be acid hyaluronidases, and they are active in the most acidic conditions, with a pH less than 4. However, the wild-type protein from crude venom tolerated a broader pH range. This protein was also active in the neutral pH, with activity in a range of pH 2 to 7. This range was observed for venom hyaluronidase from wasps, including the Thai banded wasp (*V. tropica*) [27]. In general, the wasp venom hyaluronidase enzymes exhibit maximal enzymatic activity at pH 5-6 [1]. *V. affinis* venom still has hyaluronidase activity at neutral pH (pH about 7). These results demonstrated that the activity of this wasp venom may act as a spreading factor under normal human physiological conditions, where the pH is near 7.0, which aids in venom toxin diffusion into victim tissues [46, 52-55].

The enzymatic activity of the recombinant wild-type VesA2 decreased rapidly from pH 2 to 3 and completely lost all activity at pH 4. This indicated that rVesA2 is an extremely active enzyme at strongly acidic pH. For the wild-type rVesA2, although no substitutions had been made, the proteins shifted their optimal pH from a neutral to an acidic pH. Glycosylation has been identified as a factor responsible not only for increasing protein stability and causing allergenic properties but also for influencing the catalytic activity, pH optimum and thermal stability of enzymes to different extents [56, 57]. The recombinant *E. coli* expression system is non-glycosylating and is mostly used to obtain the high-yield recombinant protein that is expressed, which shifts the optimal pH of rVesA2 to an acidic pH. Otherwise, the activity is decreased at a neutral pH. Therefore, the pH shift to neutral pH or basic pH may result in the loss of hyaluronidase activity in wild-type rVesA2 [58]. However, the optimal temperature of the *V. affinis* venom hyaluronidase was lower than that of other venom hyaluronidases [1, 35]. These wasps still had 50% hyaluronidase activity at 37-60 °C, which is usually found in other venom hyaluronidases [35, 55].

## Conclusion

Hyaluronidase enzymes are interesting since their application** **is varied. The enzyme originating from wasp venom was characterized and expressed for further studies. A recombinant *E. coli*-based expression system can be used for up-scale production due to its overexpression capabilities. However, refolding steps are required for the recovery of the enzyme activity. The rVesA2 wild-type enzymes showed the highest activities at a strongly acidic pH, whereas those from crude venom showed high activity at a more neutral pH. The mutant protein, with double point mutations at the catalytic sites, completely loses the enzymatic activity. These characterizations could be useful for any variety of applications.

### Abbreviations

3' RACE: 3' rapid amplification of cDNA ends; 3' UTR: 3' untranslated region; CCD: cross-reactivity carbohydrate determinant; HA: hyaluronic acid; IPTG: isopropyl-β-D-thiogalactopyranoside; PCR: polymerase chain reaction; pI: isoelectric point; rVesA2: recombinant *Vespa affinis* hyaluronidase; Trx: thioredoxin; VesA2: *Vespa affinis* venom hyaluronidase
